# A Novel Method for Single Sample Multi-Axial Nanoindentation of Hydrated Heterogeneous Tissues Based on Testing Great White Shark Jaws

**DOI:** 10.1371/journal.pone.0081196

**Published:** 2013-11-19

**Authors:** Toni L. Ferrara, Philip Boughton, Eve Slavich, Stephen Wroe

**Affiliations:** 1 Evolution and Ecology Research Centre, School of Biological, Earth and Environmental Sciences, The University of New South Wales, Sydney, New South Wales, Australia; 2 Biomedical Engineering, AMME School, The University of Sydney, Sydney, New South Wales, Australia; 3 Department of Orthopaedic Surgery, St George Clinical School, The University of New South Wales, Kogarah, New South Wales, Australia; 4 Zoology Division, School of Environmental and Rural Sciences, University of New England, Armidale, New South Wales, Australia; Dalhousie University, Canada

## Abstract

Nanomechanical testing methods that are suitable for a range of hydrated tissues are crucial for understanding biological systems. Nanoindentation of tissues can provide valuable insights into biology, tissue engineering and biomimetic design. However, testing hydrated biological samples still remains a significant challenge. Shark jaw cartilage is an ideal substrate for developing a method to test hydrated tissues because it is a unique heterogeneous composite of both mineralized (hard) and non-mineralized (soft) layers and possesses a jaw geometry that is challenging to test mechanically. The aim of this study is to develop a novel method for obtaining multidirectional nanomechanical properties for both layers of jaw cartilage from a single sample, taken from the great white shark (*Carcharodon carcharias*). A method for obtaining multidirectional data from a single sample is necessary for examining tissue mechanics in this shark because it is a protected species and hence samples may be difficult to obtain. Results show that this method maintains hydration of samples that would otherwise rapidly dehydrate. Our study is the first analysis of nanomechanical properties of great white shark jaw cartilage. Variation in nanomechanical properties were detected in different orthogonal directions for both layers of jaw cartilage in this species. The data further suggest that the mineralized layer of shark jaw cartilage is less stiff than previously posited. Our method allows multidirectional nanomechanical properties to be obtained from a single, small, hydrated heterogeneous sample. Our technique is therefore suitable for use when specimens are rare, valuable or limited in quantity, such as samples obtained from endangered species or pathological tissues. We also outline a method for tip-to-optic calibration that facilitates nanoindentation of soft biological tissues. Our technique may help address the critical need for a nanomechanical testing method that is applicable to a variety of hydrated biological materials whether soft or hard.

## Introduction

Application of nanoindentation in biological studies has been hindered by the lack of suitable protocols for testing materials with high water content [1]–[3]. Mechanical properties of tissues have been shown to vary significantly with hydration state [4]–[11]. Several methods have therefore been developed in an attempt to maintain tissue hydration [1], [4], [9]–[17]. Despite the development of these methods, maintaining the hydration state of tissues during mechanical testing remains a significant challenge [3].

**Figure 1:**
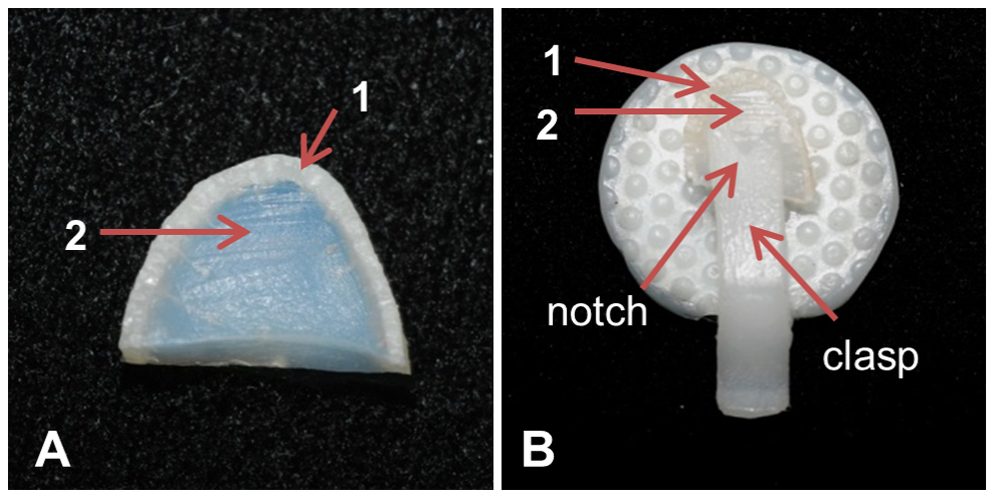
Preparation of the “dry” bull shark sample. An arch-shaped wedge of cartilage (A) possessing both mineralized (1) and non-mineralized layers (2) was held in place with the clasp of a clip (B). The “V” shaped notch cut into the clasp (B) maintained equal pressure on both sides of the sample during nanomechanical testing and assisted with positioning the sample under the microscope.

As with many biological materials [18], the mechanical properties of shark jaw cartilage are poorly known [19]. This paucity of data is partially due to the difficulty of testing this and other biological tissues. The jaw cartilage of sharks is a hydrated tissue that possesses a unique heterogeneous arrangement of both mineralized (hard) and non-mineralized (soft) layers [20]. This arrangement consists of a thin, mineralized cortex comprised of a series of blocks (tesserae) that overly a non-mineralized core (see Figure 1) that is primarily composed of water [19], [20]. Shark jaw cartilage therefore represents a material that is intermediate between bone and cartilage because it possesses a non-mineralized phase similar to hyaline cartilage and a calcified phase absent from the cartilage of other groups [19], [20].

Examination of mechanical properties using standard tensile or compression tests requires samples of uniform cross-sectional area to calculate stress in the sample. Thus, previous mechanical testing of shark cartilage has focused primarily on testing vertebral centra (the circular, central portion of the vertebrae) or machine-cut core samples through the non-mineralized layer of jaw cartilage [21]–[24]. Although bulk material properties are available for a very limited number of shark species [19], [25], the shape of the jaws and the very thin layer of mineralized cartilage prohibits obtaining values for different cartilage layers using a material testing system (MTS). Shark jaw cartilage is therefore an ideal material for developing a method for nanoindentation of heterogeneous tissues as it is a hydrated composite of both mineralized (hard) and non-mineralized (soft) tissue with a geometry that limits testing in a MTS.

The aim of this work is to develop a multi-axial, nanomechanical method suitable for testing and comparing both layers of cartilage from a single, small sample of hydrated shark jaw cartilage. We examined two nanomechanical properties: Young’s modulus (a measurement of stiffness) and indentation hardness. The jaws of the great white shark (white shark; *Carcharodon carcharias*) were chosen for this study as obtaining tissue samples is often difficult because these sharks are a protected species [26]. Similar scenarios may also occur when testing other tissues and biomaterials that can be valuable, scarce or minute in size, such as samples obtained from humans, pathological tissues, explanted implants, endangered species, or fossils [1], [3], [18], [27]. Furthermore, tissues are rarely homogeneous or isotropic, and tissue mechanics are also known to vary with anatomical position [28]. Obtaining multidirectional nanomechanical properties from single locations will therefore be highly advantageous.

## Methods

### 2.1. Overview

Since only one sample of jaw cartilage from the great white was available, nanoindentation was initially performed on samples from a bull shark (*Carcharhinus leucas*) to develop a suitable hydration method for testing both mineralized and non-mineralized jaw cartilage. Bull shark samples were tested in two ways: in the first experiment (called “dry”) no attempt was made to hydrate samples while in the second experiment (called “wet”) novel experimental techniques were used to maintain hydration. These testing methods are outlined in sections 2.2 through 2.7. Sections 2.8 through 2.10 outline how results from bull shark cartilage testing were used to develop a multi-axial testing method for nanoindentation of great white jaw cartilage. All specimens for this study were provided with full ethics approval from the Department of Primary Industries (DPI) Fisheries, Cronulla, New South Wales, Australia and in accordance with UNSW Animal Care and Ethics Committee guidelines. No animals were sacrificed in this study.

### 2.2. Sample preparation: bull shark

The jaws were removed from the head of a male bull shark (145 cm in total length; NSWDPI # AB020311-2) which was obtained from DPI Fisheries, Cronulla. The head was removed from a freshly killed animal by commercial fishers and kept frozen prior to dissection. An arch-shaped section of cartilage approximately 1 mm thick and 5 mm in height was cut in the frontal plane of the jaw immediately posterior to the final tooth file on both the right and left sides of the lower jaw using a razor blade. As mechanical properties may vary according to anatomical location [28], both samples were removed from this location on opposite sides of the jaw to standardize position and thus facilitate comparisons of nanomechanical properties between sections treated “dry” and “wet” (see below). This position was also chosen as it corresponds to high values for von Mises stress calculated in a finite element analysis of the jaws of the great white [29]. All muscle and connective tissue (including the perichondrium) were removed from the jaws exposing the mineralized layer of cartilage in this area prior to cutting the sections.

### 2.3. Preparation of “dry” and “wet” bull shark specimens

**Figure 2:**
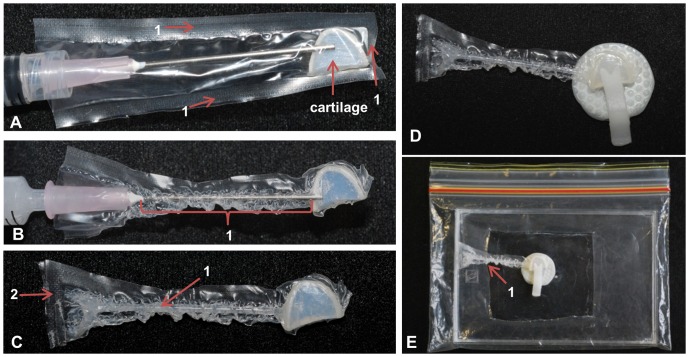
Sample preparation in an enveloping pouch. A plastic bag was heat sealed along 3 sides (1A) to form a pouch. A section of cartilage (A) was inserted into the pouch. A syringe filled with a hydrating medium was placed in the pouch with the opening of the needle positioned beneath the sample (A). The pouch was then heat-shrunk with a hair dryer allowing the pouch to conform to the sample and the needle, forming a channel (1B) along the length of the needle. The hydrating medium (1C) was injected into the channel as the needle was carefully withdrawn. The end of the channel was heat sealed and cut forming a tail (2C). A small rectangular window (not shown) was cut in the pouch to expose the surface for testing and the sample and pouch were then placed in a clip (D). The sample was then placed in a hydrating chamber (E) with the tail of the pouch tucked beneath the rectangular opening of the chamber (1E) to facilitate nanomechanical testing.

The sample from the left side of the jaw was prepared without further modification as a “dry” specimen (Figure 1) while the sample from the right side of the jaw was tested “wet” in a pouch (Figure 2). Bull shark samples were tested “wet” in pouches to minimize dehydration during testing. The pouch was created by measuring the base of a polyethylene plastic sandwich bag to the approximate height of the sample then heat sealing the bag just short of the measured distance on a diagonal at approximately 30° to form a trapezoidal shaped pouch (Figure 2 A). A trapezoidal shape was chosen to ensure a tight fit between the wedge–shaped cartilage sample and the pouch while maintaining an opening at the base of the pouch wide enough to fit a syringe.

A 3 mL syringe was filled with a hydrating medium (*Hydrochond*, Biomedic, Sydney, Australia) and inserted into the pouch with the opening of a needle (1.2×38 mm; Becton Dickinson, Singapore) placed beneath the base of the sample (Figure 2 A). The sample and needle were then submerged in ice for several minutes to cool the sample. Care was taken to ensure the sample did not get wet. A hair dryer with a digital temperature indicator was set to between 90–93°C. The pouch (containing both the sample and needle) was then heat shrunk with warm air from the hair dryer for approximately 3 seconds until the polyethylene conformed to the chilled sample and needle (Figure 2 B). This created a channel in the pouch along the length of the needle (Figure 2 B). Immediately after the polyethylene pouch was shrunk to the desired shape it was re-submerged in ice for several minutes while ensuring the sample did not get wet. Although the heat shrinking process is unlikely to overly warm the specimen, keeping the sample on ice before and after shrink-wrapping helped minimize sample temperature elevation. The hydrating medium was then slowly injected beneath the sample and into the channel as the syringe was carefully withdrawn (Figure 2 C). Placement of the needle beneath the sample prior to heat shrinking allowed a thin film of the hydrating medium to cover the base of the sample. To further protect against the sample losing water, the end of the channel was then heat sealed forming a fully enclosed pouch (Figure 2 C). Care was taken to ensure the hydrating medium was not squeezed onto the sample test surface.

A small rectangular window approximately 2 mm^2^ was cut from the apex of the arch of the sample into the pouch with a scalpel blade (number 11 surgical blade; Swann- Morton, Sheffield, England) using a desk mount magnifying glass with light attachment to view the sample. The sample (in its pouch) was then carefully placed in the clip (Figure 2 D), so the edge of the pouch window (not shown) was aligned with the “V” shaped notch in the clasp (see Figure 1) while ensuring that the hydrating medium did not spread onto the testing surface. The position of the window relative to the notch facilitated the alignment of the sample under the microscope for testing. Care was taken to determine the tip-to-optic boundary of the pouch testing window prior to nanoindentation.

To further prevent dehydration of the sample, a hydrating chamber with humidifying atmosphere was created by placing a culture plate tray in a re-sealable plastic sandwich bag with cut rectangle opening (Figure 2 E). The hydrating chamber was placed on the magnetic stage of the nanoindenter. The sample (in its pouch and clip) was placed in the hydrating chamber through the rectangular opening taking care not to disrupt its position in the clip or cover the testing surface with hydrating medium. The tail of the pouch (Figure 2 E) was tucked under the rectangular opening of the hydrating chamber so it would not interfere with the indenter probe during testing. An isotonic solution similar to shark plasma [30] was injected through the rectangular opening of the hydrating chamber with a 10 mL syringe. Prior to placing the hydrating chamber on the stage, a thin film of liquid soap was used to coat the bottom of the tray to allow the isotonic solution to spread evenly over the base of the chamber. Addition of the isotonic solution with a syringe to the coated chamber after the sample was in place controlled the flow of the solution in the chamber and ensured that the sample was not covered with the fluid. The rectangular opening of the hydrating chamber allowed the nanoindenter probe to contact the sample and created a localized moisture environment around the sample via evaporation of the isotonic solution from the chamber.

### 2.4. Nanoindentation of bull shark jaw cartilage

Quasi-static nanomechanical properties for both the mineralized and non-mineralized layers of wet and dry bull shark jaw cartilage were determined using nanoindentation performed on a Hysitron Triboindenter (Hysitron, Minneapolis, USA) with a light microscope mounted to the indenter. Optical imaging of the sample using the microscope allowed precise control between the sample position and the indenter thus ensuring that results from each test were obtained solely from mineralized or non-mineralized layers of cartilage. A conicospherical tip with a radius of 100 µm was chosen as it was found to perform best with both rough surfaces (such as the mineralized layer) and soft tissue types (such as the non-mineralized layer; [1]).

Each indentation utilized a trapezoidal load function consisting of a linear loading segment of 10 seconds followed by a 5 second hold period at a predetermined peak force and a linear unloading segment of 10 seconds. Trapezoidal load functions have been shown to minimize the effects of creep when calculating Young’s modulus from viscoelastic materials [2], [3]. A 5 second hold period [1], was chosen to allow the indenter to settle into the cartilage prior to unloading. Bull shark samples were tested at both 100 µN and 1000 µN peak forces during the hold period. Young’s modulus, hardness and contact depth were recorded from “dry” and “wet” samples of both layers of bull shark cartilage samples. Reduced elastic modulus and hardness were determined using the compliance method of [31]. Young’s modulus for shark cartilage (*E_s_*) was determined from reduced elastic modulus (*E_r_*) using the equation: 
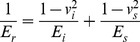



Where *E_i_* is the modulus of the indenter (1140 GPa), *v_i_* is the Poisson’s ratio for the indenter (0.07) and *v_s_* is the Poisson’s ratio for shark cartilage (0.3) determined by [24].

### 2.5. Tip-to-optic calibration

Although indium is commonly used to calibrate conical tips with a radius of up to 50 µm, this material is insufficient for calibration of larger tips. Tip-to-optic calibration was therefore determined by nanoindentation of chocolate (KitKat®) as it (compared to indium or modelling clay for example) was found to be the most reliable medium for X-Y positional calibration of 100 µm conical tips. Indentation of the KitKat left a clearly recognizable mark in the smooth, flat surface of the chocolate which, once properly calibrated, aligned with the cross hairs of the optical window after testing. Furthermore, chocolate residue is easily cleaned from the indentor tip (using manufacturer’s instructions) and KitKats are rigorously manufactured with an exceptional flat tolerance and possess fewer microscopic air bubbles (which would obscure marks made by the indenter probe) than other materials.

### 2.6. Results of bull shark jaw cartilage tested “dry”

**Figure 3:**
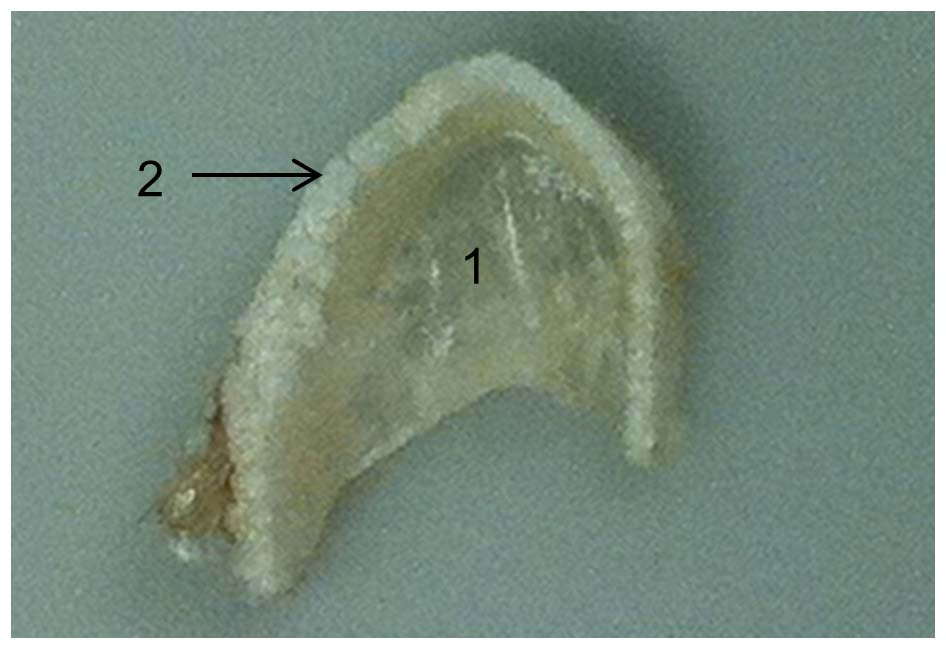
Desiccation of cartilage in the “dry” specimen. The image shows the effect of water loss if no attempt is made to maintain hydration during nanoindentation. Due to significant moisture loss in the sample, the non-mineralized layer (1) is noticeably shrivelled and paper thin. The mineralized blocks (tesserae) present in the outer layer are also visible (2).

In order to obtain nanomechanical properties for cartilage using the nanoindenter, the exact location of the sample must first be defined in *x*, *y*, and *z* axes using the microscope thus defining the testing position for the indenter. During testing, the focal plane (*z* axis) of the specimen changed rapidly due to dehydration of the sample. This rapid dehydration resulted in the sample being out of focus in less than one minute and thus prevented the completion of nanomechanical testing. Results for dry specimens could therefore only be obtained once the sample was noticeably desiccated (Figure 3). Young’s modulus (*E*), hardness (H) and contact depth (h_c_) for the mineralized layer of the dry specimen at peak forces of 100 and 1000 µN are found in Table 1. Dehydration resulted in extensive shrivelling of non-mineralized cartilage (Figure 3) prohibiting results from being obtained from this layer.

**Table 1:** Young’s modulus (*E*), hardness (H) and contact depth (h_c_) for bull shark jaw cartilage.

	Peak force	*E*	H	h_c_ (nm)
Mineralized cartilage (dry)	100	159.5 MPa	1.5 MPa	196.0
	1000	1.2 GPa	7.7 MPa	373.7
Mineralized cartilage (wet)	100	78.6 MPa	578.9 KPa	494.6
	1000	220.2 MPa	4.2 MPa	687.1
Non-mineralized cartilage (wet)	100	35.9 MPa	246.7 KPa	2555.9
	500	17.7 MPa	359.1 KPa	4117.3

### 2.7. Results of bull shark jaw cartilage tested “wet”

Unlike dry specimens, no changes in focal plane were observed during testing of the wet (i.e. in pouches) bull shark sample. Table 1 shows the results for Young’s modulus, hardness, and contact depth for mineralized and non-mineralized cartilage tested wet. Young’s modulus and hardness for both mineralized and non-mineralized cartilage were variable depending on the peak force used (Table 1). Non-mineralized cartilage could not be tested at peak forces exceeding 500 µN as the displacement limit for the depth of indentation of the Hysitron system is 5000 nm and the maximum depth recorded for tests on non-mineralized cartilage at peak forces of 500 µN was 4482.3 nm. Nanoindentation of non-mineralized cartilage at peak forces of 500 µN were therefore frequently terminated due to displacement errors during testing, suggesting that this is the maximum peak force for this type of cartilage.

Testing errors were not observed during nanoindentation of non-mineralized cartilage at a peak force of 100 µN. Based on these results, a peak force of 100 µN was chosen to facilitate testing and comparison of nanomechanical properties of mineralized and non-mineralized great white shark jaw cartilage.

### 2.8. Sample preparation: great white shark

The jaws were removed from the head of a male great white shark (2.45 m total length; NSWDPI-SMP-WAT061210), that was obtained from a dead specimen that was caught in the shark nets by the NSW Shark Meshing (Bather Protection) Program. An arch-shaped sample of cartilage measuring approximately 2 mm thick and 7 mm in height was cut in the frontal plane of the upper jaw immediately posterior to the final tooth file jaw using a razor blade. All muscle and connective tissue (including the perichondrium) was removed from the jaws exposing the mineralized layer of cartilage in this area prior to cutting the sample. Based on the results of bull shark tests, the great white cartilage sample was tested in a pouch as outlined in Section 2.3 to minimize dehydration (and see Figure 2).

**Figure 4:**
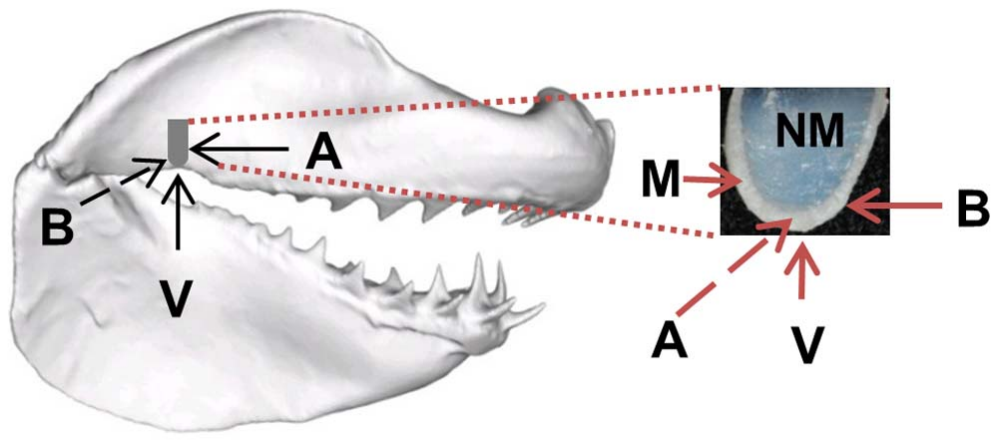
Diagram of the cartilage sample removed from the great white shark jaw for nanomechanical testing. The sample is shown *in situ* (grey outline) and removed, rotated and enlarged to show both mineralized (M) and non-mineralized (NM) layers of cartilage. Arrows indicate the anterior (A), buccal (B) and ventral (V) surfaces of the sample relative to the jaw. Surfaces are defined as follows: anterior surface of the sample corresponded to the frontal plane (anteroposterior axis) of the jaws; the buccal surface of the sample corresponded to the sagittal plane (mediolateral axis) of the jaws; the ventral surface of the sample corresponded to the transverse plane (longitudinal axis) of the jaws. Dashed arrows indicate directions perpendicular to the page.

**Figure 5:**
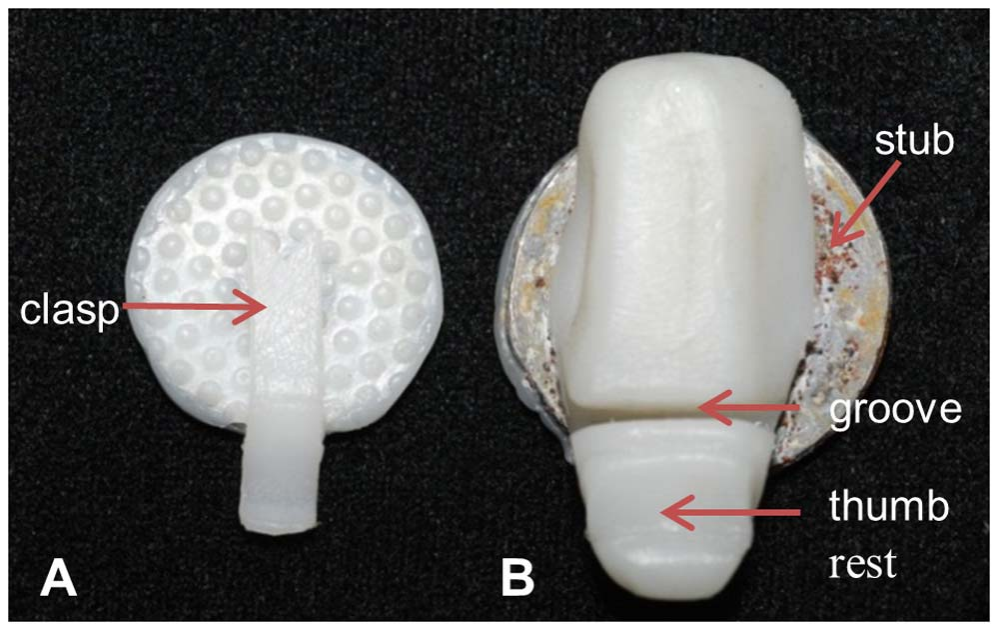
Clips purpose built for nanomechanical testing. Clips were moulded using thermoplastic aliphatic polyester encompassing a ferromagnetic steel stub which is visible in Clip B. The steel stub allowed the clips to adhere to the magnetic stage of the nanoindenter. In Clip A, the sample was held in place with a clasp (A) and a textured surface was moulded into the base of the clip to further minimize movement of the sample during testing. Samples were loaded into the groove of Clip B by depressing the thumb rest. The width of the groove in the flexible clip was chosen to accommodate samples between 1–3 mm in thickness and held the cartilage firmly in place without compressing the sample. The anterior surface of the sample was tested using Clip A, while the buccal and ventral surfaces were tested by rotating the sample in Clip B. Only one sample was required to complete multi-axial testing on both layers of cartilage because the sample was not bonded to the stub and could be easily rotated into different positions using the clips.

**Figure 6:**
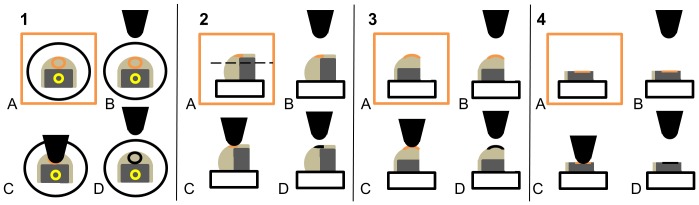
Schematic of multi-axial nanomechanical testing. In panel 1A, the sample of cartilage with mineralized (light grey) and non-mineralized (dark grey) cartilage is placed flat in Clip A (black circle) under a microscope (orange box). The testing position (on mineralized cartilage) is defined by the optical cross hairs (orange circle, A) of the microscope and this position is coupled to the indenter position (orange circle, B). Indentation occurs at the position defined by the optical cross hairs (C, D). Each indentation is a repeat of steps A-D and numerous indentations are performed on the sample. Upon completion of testing mineralized cartilage, the optical cross hairs are moved to a location on non-mineralized cartilage (yellow circle, 1A) and steps in Panel 1A-D are repeated to test the anterior surface of this layer (not shown). In Panel 2, the cartilage sample is placed in Clip B (black box) and the indentation sequence (A-D) is repeated numerous times to test the buccal surface (orange) of mineralized cartilage. The upper portion of the sample is removed (dashed line, Panel 2A) and the sequence is subsequently repeated to test the buccal surface of non-mineralized cartilage (not shown). In Panel 3 the sample is rotated in Clip B to test the ventral surface (orange) of mineralized cartilage. The upper portion of the sample was then removed to test the ventral surface (orange) of non-mineralized cartilage (Panel 4A-D). All tests were conducted in a hydrating chamber (not shown).

To examine if nanomechanical properties varied with direction, the great white sample (in its pouch) was tested in three perpendicular directions relative to the jaw. The orientation of the cut section of cartilage relative to the jaw was recorded for the great white sample (Figure 4) prior to placing the sample in its pouch. The anterior, buccal and ventral surfaces of the sample (Figure 4) were chosen as they are in perpendicular planes (i.e. to test for orthotropy). Thus a total of 6 tests (3 directions for each layer of cartilage) were conducted on the great white sample. Each test consisted of several indentations (see Table 2). Two specially designed clips were constructed to allow all 6 tests to be conducted on a single sample (Figure 5). All tests were competed in a hydrating chamber (shown in Figure 2 E) with the sample in either Clip A or B (Figure 5; and see Figure 6).

**Table 2:** Young’s modulus and hardness for great white shark jaw cartilage in three directions.

	Buccal	Ventral	Anterior
Young’s Modulus Mineralized	229.1; 21	130.8; 32	118.8; 32
Young’s Modulus Non-mineralized	9.974; 6	10.27; 12	15.52; 10
Hardness Mineralized	1.457; 21	1.900; 32	1.856; 32
Hardness Non-mineralized	0.296; 6	0.194; 12	0.460; 10

Note: Numbers represent the mean (in MPa) followed by the number of indentations.

### 2.9. Multi-axial, biphasic nanoindentation of a single sample of great white jaw cartilage

As determined from bull shark tests, quasi-static nanomechanical properties (Young’s modulus; hardness) for both the mineralized and non-mineralized layers of great white jaw cartilage were determined using nanoindentation performed with a Hysitron Triboindenter using a conical tip with a radius of 100 µm. Each indentation consisted of a linear loading segment of 10 seconds followed by a 5 second holding period at a peak force of 100 µN (to facilitate comparisons between layers) and a linear unloading segment of 10 seconds.

Young’s modulus and hardness were obtained from the anterior, buccal and ventral surfaces of both mineralized and non-mineralized layers. To test the anterior surface of the sample (Figure 6, Panel 1) the sample (in its pouch, not shown) was placed flat in Clip A (Figure 5; represented by a black circle in Figure 6). A small rectangular window approximately 2 mm^2^ was cut from the apex of the arch of the sample into the pouch with a scalpel blade. After nanoindentation was completed for both layers of cartilage on the anterior surface, the sample (in its pouch, not shown) was rotated and placed in Clip B (Figure 5; represented by a black box in Figure 6) to test the buccal surface. A rectangular window approximately 1 mm in width and 3 mm in length was cut in the pouch with a scalpel blade exposing the buccal surface for testing. Once nanoindentation data was acquired for the buccal surface of the mineralized layer (Figure 6, Panel 2), the upper portion of the sample (and the pouch) was cut approximately 2 mm beneath the lowermost boundary of the mineralized layer with a scalpel blade and removed to expose the buccal surface of the non-mineralized layer (Figure 6, Panel 2, dashed line). After testing on the buccal surface of the non-mineralized layer was complete, the remaining sample was sealed in a new pouch (not shown) and then rotated in Clip B to expose the ventral surface of the mineralized layer of the sample for testing (Figure 6, Panel 3). A rectangular window (approximately 1 mm in width and 3 mm in length) was cut in the pouch to test the mineralized layer in this direction. Upon completion of testing the buccal surface of the mineralized layer (Figure 6, Panel 3) the sample and pouch were cut approximately 2 mm below the lowermost boundary of the mineralized layer of cartilage to expose the buccal surface of the non-mineralized layer for testing (Figure 6, Panel 4). Samples and windows were cut using a number 11 scalpel blade (Swann- Morton, Sheffield, England), a ruler and with the aid of a mounted desk top magnifying glass.

### 2.10. Statistical analysis

To examine if there were directional differences in nanomechanical properties of mineralized and non-mineralized great white jaw cartilage separate statistical models were fitted for Young’s modulus and hardness. Complete weighted generalized least squares (GLS) regression models were fitted on the log transform of either Young’s modulus or hardness using the restricted maximum likelihood method with the package *nlme* [32] in R 2.13 [33]. The model was fitted with a variance function allowing for heteroscedasticity (different variances in the mineralized and non-mineralized layers [34]). Factors and levels for each model were “Layer” (2 levels: mineralized; non-mineralized cartilage) and “Direction” (3 levels: ventral surface; anterior surface; buccal surface). Significance of factors and interactions were determined by using ANOVAs of the GLS models. Significance was determined using the Bonferroni correction (α  =  0.05). If a significant directional effect was observed in the GLS model contrasts were constructed to compare each pair of directions (Buccal:Ventral; Buccal:Anterior; Ventral:Anterior) to examine the underlying cause of the effect.

## Results

### 3.1. Young’s modulus and hardness of great white jaw cartilage

**Figure 7:**
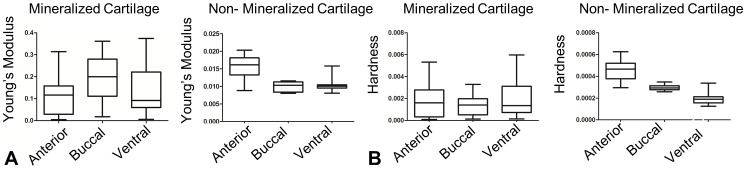
Boxplots. Boxplots show variation in Young’s modulus (A) and hardness (B) for mineralized and non-mineralized great white shark jaw cartilage in different directions. Axes are in gigapascals (GPa). Whiskers on boxplots represent minimum and maximum values.

Hydration of the great white sample was maintained for the 8 hours it took to complete all 6 tests. Boxplots (Figure 7), average values for nanomechanical properties of great white jaw cartilage (Table 2) and results of contrasts testing directional differences (Table 3) show that there were significant directional differences detected in nanomechanical properties for both mineralized and non-mineralized white shark jaw cartilage. Although there was no significant difference detected with direction in hardness for the mineralized layer, the buccal surface was significantly stiffer (i.e. Young’s modulus) than the anterior surface (Figure 7; Tables 2 and 3) in this layer. In the non-mineralized layer there was marginal evidence (α  =  0.1) to suggest that the anterior surface is stiffer than the ventral surface and strong evidence (α  =  0.01) that the anterior surface was harder than the ventral surface (Figure 7; Tables 2 and 3).

**Table 3:** Bonferroni adjusted *p*-values for results of contrasts testing directional differences.

	Buccal:Ventral	Ventral:Anterior	Buccal:Anterior
Young’s Modulus Mineralized	.006	.373	.000
Young’s Modulus Non-mineralized	.872	.003	.009
Hardness Mineralized	Not significant		
Hardness Non-mineralized	.011	**.000**	.020

Note: Numbers that are in bold or underlined are significant at α  =  0.05 and 0.1, respectively; “Not significant” indicates contrasts were not performed since no directional effect was observed.

### 3.2. Structure of great white jaw cartilage

**Figure 8:**
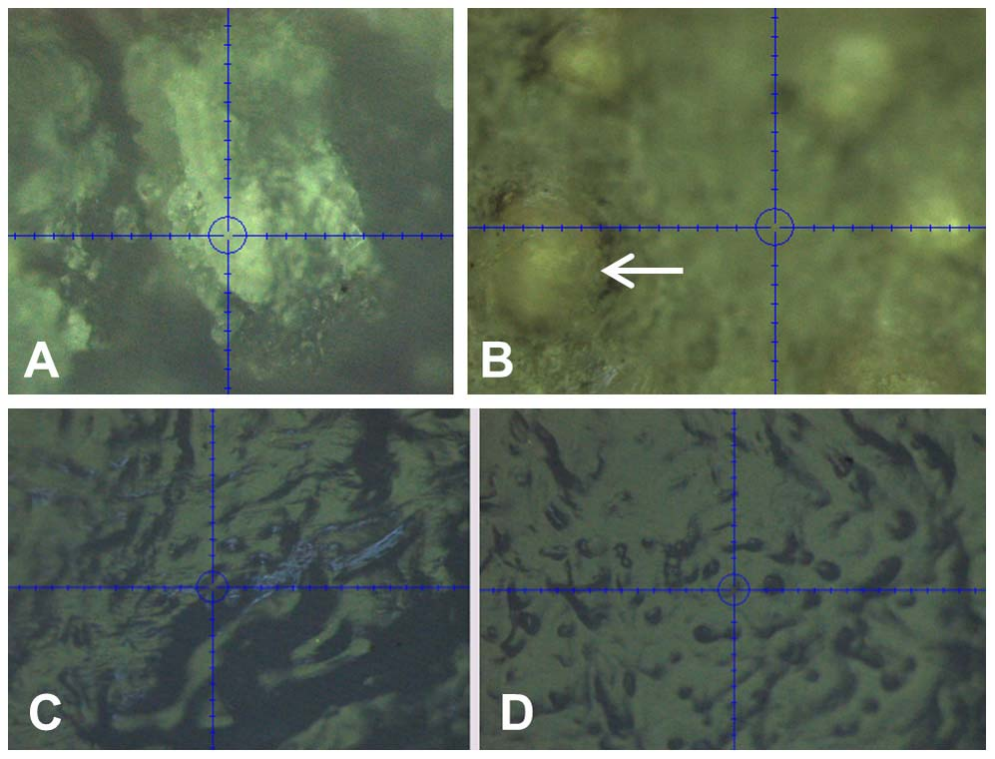
Optical Images from the Hysitron Triboindenter of mineralized (A, B) and non-mineralized (C, D) great white shark jaw cartilage. The image of the anterior surface of mineralized cartilage (A) shows the optical cross hairs (blue) on a block of mineralized cartilage. The image of the ventral surface (B) shows numerous nodules (e.g. white arrow) present in the jaw cartilage. Images C and D are both from the ventral surface of the same sample of cartilage and show differences observed in topology in the non-mineralized layer.

Distinct “nodules” were clearly visible in mineralized great white jaw cartilage (Figure 8 B). Indentations were performed on nodules in the ventral surface of mineralized cartilage with Young’s modulus ranging from 5.860 – 34.54 megapascals (MPa) and hardness ranging from 0.134 – 0.394 MPa.

Although the non-mineralized layer was more homogeneous in appearance than the mineralized layer, this layer still displayed differences in topology (Figure 8) and nanomechanical properties (Table 3; Figure 7). Fewer indentations were conducted on non-mineralized cartilage (Table 2) as fewer surface structures were observed in this layer compared to mineralized cartilage.

## Discussion

### 4.1. Great white jaw cartilage

Our study presents the first analysis of nanomechanical properties from the jaw cartilage of the great white shark. Multidirectional variation in Young’s modulus and hardness was observed in both mineralized and non-mineralized great white jaw cartilage (Table 3; Figure 7). Nodules of low stiffness and hardness (similar to properties for non-mineralized cartilage; Table 2) were observed in the mineralized layer of great white jaw cartilage but not in bull shark jaw cartilage. As results may vary due to methodology [18], [35], our results recommend that further nanomechanical studies of chondrichthyan cartilage utilize a peak force of 100 µN to facilitate comparisons between mineralized and non-mineralized layers. Variation in biological tissue structure and mechanical properties are often attributed to function [28], [36]–[40]. However, as there is little information on the nanostructure of shark jaw cartilage [20], [40], it remains to be determined if and how nanostructural differences in mineralized and non-mineralized shark jaw cartilage relate to feeding and if there are differences with ontogeny [29].

Young’s modulus for the mineralized layer of elasmobranch cartilage has been reported as 2.00 GPa [41] and 4.05 GPa [24]). These values are within the lower range for bone (1–20 GPa; [3]). In contrast, mean Young’s modulus values for the mineralized layer of great white jaw cartilage in this study ranged from 0.12 to 0.23 GPa (Table 2), at least an order of magnitude lower than previously reported values. Thus, in comparison to stingray data (round stingray, *Urobatis halleri;* [24]) shark jaw cartilage may be less stiff than previously considered.

### 4.2. Utility of method

Our study provides a novel method for obtaining multidirectional, nanomechanical properties from both layers of hydrated great white jaw cartilage using only a single sample. Custom clips used in this study were built to clasp and immobilize tissue samples ranging in size from less than 1 mm to 3 mm in width. Samples can be re-used in multiple tests to obtain multidirectional nanomechanical properties (Figure 6) because samples are placed in specially designed clips (Figure 5) and not super-glued to a stub (as is standard practice). Our sample-sparing technique is therefore suitable for situations when samples are limited, valuable, or rare (e.g. fossils; pathological tissues; endangered species [1], [3], [18], [27]). Our technique may therefore be of benefit in examining anisotropic nanomechanical properties for an array of both soft and hard hydrated, heterogeneous, hierarchical biological materials. Novel nanoindentation procedures suitable for testing hydrated biological materials are crucial for further advances in the study of tissue mechanics, biomedical engineering and tissue engineering [1]–[3], [18], [42]–[44].

The indentation sequence outlined in Figure 6, A-D takes approximately 5 minutes. The dry bull shark sample was sufficiently out of focus to prohibit testing in less than one minute. Although dehydration would be expected in samples with a high surface to volume ratio (such as the ones used in this study), the inability to maintain a usable focal plane during testing of dry bull shark samples for such a short period of time was unexpected. Our method maintained hydration as evidenced by minimal changes in focal plane during testing for the 8 hours required to complete all 6 tests (each consisting of numerous indentations) on the great white sample. In addition, hydration is applied from a hydrating medium (*Hydrochond*) that is stored in the channel of the pouch. The dimensions of this channel can be altered to store additional hydrating medium for experiments of longer duration, such as overnight. Furthermore, several studies maintain hydration in samples by submerging the sample in liquid [4], [9], [10], [12]–[15]. This technique may complicate testing as specialized tips are required and application of fluids interferes with visualization of the sample, making it difficult to define the test site [1], [3], [15]. These complications are avoided using our method, as a standard conical tip is used and hydration is applied to the base of the sample, and is thus far removed from the testing position.

Improved techniques for tip-to-optic calibration during nanoindentation are critical for testing soft biological tissues [1]. Tip-to-optic calibration was completed via indentation of a soft chocolate with a smooth, flat surface. This method is an advance over previous calibration methods as spherical tips (including those larger than 20 µm) required for testing soft tissue [1], [3], [16], [45], [46] can be directly calibrated using this technique rather than relying on calibration using a Berkovich tip prior to switching to the spherical tip, which may inadvertently change the alignment leading to erroneous results [1].

Examination of the mechanical properties of an array of largely unknown vertebrate tissue types may offer new insights into biomedical and biomimetic designs [18], [38], [47]. Currey [18] has stated that: “There is a pressing need for an examination of some material properties of a whole variety of bones, always using exactly the same testing method, for instance nanoindentation of wet material, so that firm comparisons can be made.” Currey [18] included shark cartilage in this assessment. Our method may be applicable to numerous biological materials because it maintained hydration in a single small sample during multidirectional nanomechanical testing, recommends an optimum loading rate for analysis of both mineralized and non-mineralized tissue, and outlines a tip-to-optic calibration method suitable for soft tissues. Defining a peak force (100 µN) suitable for comparisons of soft and hard tissues is necessary given that displacement errors are common during nanoindentation of soft, hydrated biological tissues [45]. Examination of a range of animal tissues using our method may therefore assist in further understanding of form, function and evolutionary pathways of biological systems, as well as future innovations in biomimetic and biomedical device designs.
